# Returning the Favor? Feeling Obliged and Reported Participation in Discretionary Safety Activities

**DOI:** 10.3389/fpsyg.2021.674110

**Published:** 2021-08-31

**Authors:** Julie Laurent, Nik Chmiel, Isabelle Hansez

**Affiliations:** ^1^Human Resources Development Unit, University of Liège, Liège, Belgium; ^2^Department of Psychology and Counselling, University of Chichester, Chichester, United Kingdom

**Keywords:** social exchange theory, anticipatory reward, felt obligation, safety participation, perceived organizational support, organizational support theory

## Abstract

Recent research has shown that the reported participation of employees in voluntary safety activities is related to the prevention of accidents and injuries. Encouraging such participation, then, is beneficial to organizations. A key question, therefore, is why employees should choose to report that they engage in such activities: what is their motivation given such activities are not compulsory? We used social exchange theory (SET) and organizational support theory (OST) to develop a model linking perceived organizational support to reports of safety participation. SET postulates that the benefits given (by an organization) are reciprocated with potential benefits to the giver as a result. OST emphasizes that feeling obliged is a key part of why people reciprocate the perceived support they get from their organization. Voluntary safety activities have the potential to benefit an organization, so for the first time, we test whether there is a link between perceived organizational support and the reported participation of employees in such activities, and whether the relationship is mediated by felt obligation. We also test whether another key SET motivation to reciprocate, the anticipated reward, is involved in mediating the relationship. A structural equation model with a sample of 536 workers from a Belgian public company, involved in the production and distribution of safe drinking water and in waste water treatment, supported the hypotheses of the authors. The model showed that felt obligation mediated the relationship between perceived organizational support and safety participation reports, and that the anticipatory reward, in the form of perceptions that management was committed to safety, also mediated the relationship between perceived organizational support and safety participation reports. These processes were shown to be separable from employee job engagement and employee perspectives on whether or not voluntary safety activities were part of their job. The findings add to the understanding of why employees choose reported participation in voluntary safety behaviors and also, add to the literature on OST by demonstrating for the first time the involvement of felt obligation and perceived management commitment to safety as mediators between outcomes and perceived organizational support.

## Introduction

Employees choosing to take part in voluntary safety activities, those beyond their job such as volunteering for safety committees or speaking out about safety when not required to do so, can be seen to be beneficial to their organization. Employee reports of their involvement in such activities have been shown to prevent accidents and injuries (Curcuruto et al., 2015), and have also been shown to decrease involvement in self-reported safety violations and increased future compliance with safety rules and procedures (Neal and Griffin, 2006; Chmiel et al., 2017; Laurent et al., 2018), which are associated with reductions in injuries and accidents (Clarke, 2006).

A key question, therefore, is why do employees report that they engage in such discretionary activities: what is their motivation to be involved, given such activities are not compulsory? It seems straightforward to propose that it has something to do with them as people, and/or that it is a function of how they relate to their organization.

Regarding the person, it may be that some employees are more altruistic than others. Recent research has provided some support that aspects of the personality of employees indeed predict safety behaviors, such as discretionary types of behavior (Beus et al., 2015), and that altruism is a predictor of employee reports of discretionary safety behaviors, but possibly only those directed toward individuals rather than the organization (Laurent et al., 2020).

In this article, we explore the second alternative: that the relationship between an employee and their organization plays a part in motivating reports of discretionary safety behaviors. Specifically, we use social exchange theory (SET) and organizational support theory (OST) to develop a model linking perceived organizational support to reports of participation in discretionary safety activities, and we examine, for the first time, whether felt obligation on the part of employees is important in predicting such reports. We also examine whether the relationship may be partly due to employees changing their perspective on what should be included as part of their job. Furthermore, we consider two other potential motivators from a social exchange perspective, namely, anticipatory reward and job engagement. In examining these issues, we aim to advance and test a structural model that can account for the association of these factors simultaneously. We use structural equation modeling (SEM) techniques to achieve this aim.

### Perceived Organizational Support and Participation in Discretionary Safety Activities^1^

Social exchange theory is considered one of the most enduring and widely used conceptual frameworks for understanding workplace behavior (Cropanzano et al., 2017). Central to SET is the idea that social exchange involves a series of interdependent and contingent interactions that generate reciprocation through obligations and mutual commitment rather than involving explicit bargaining, and that imply employees are likely to match goodwill and helpfulness toward the party with whom they have a social exchange relationship (Cropanzano and Mitchell, 2005). This idea has been used to frame understanding of the relationships between work-based support and resources and a variety of organizational behaviors, including the relationship between discretionary safety citizenship behaviors and leader-member exchanges (LMXs) (Hofmann et al., 2003) and between reports of involvement in discretionary safety activities and job resources (Laurent et al., 2018).

Here, the authors are concerned, in particular, with potential reciprocation toward the organization, so they turned to organizational support theory (Eisenberger et al., 1986) to help them develop the model.

Perceived organizational support is the construct at the heart of organizational support theory, which itself is rooted in social exchange theory. POS has been defined as a set of general beliefs concerning “the extent to which employees believe their organization values their contributions and cares about their well-being” (Eisenberger et al., 1986, p. 501). Essentially, the theory predicts that employees who perceive their organization values and cares for them are motivated to reciprocate in ways valued by the organization. A number of studies have shown that POS is related to a variety of outcomes as would be expected from the theory, such as absenteeism (Eisenberger et al., 1986) and organizational citizenship (Witt, 1991). However, the evidence relating to discretionary safety behaviors is sparse. A study by Hofmann and Morgeson (1999) showed a positive relationship between POS and feeling more comfortable and open about upward safety communication among group leaders. Open communication around safety is discretionary, and fits into a more general category of discretionary safety behaviors. More recently, Reader et al. (2017) showed that activities supporting workforce health increased perceptions of organizational support, which resulted in more safety citizenship behaviors through increased levels of commitment to the organization. We argue that such behaviors may be seen by employees as helpful to organizations where safety is valued. We would expect, therefore, that in an organization that values safety:

H1: There will be a positive relationship between POS and reported participation in discretionary safety activities.

### Felt Obligation as a Mediator

Organizational support theory draws on a proposal outlined by Gouldner (1960) that reciprocity is a universal norm and the return of favorable treatment is obliged. Thus, Eisenberger et al. (2001), and Baran et al. (2012) reasoned that POS would elicit felt obligation from employees to care about the welfare of the organization and to help the organization reach its objectives; which they could do through greater affective commitment to the organization and greater efforts to help the organization. Results from Eisenberger et al. showed, consistency with the theory, that felt obligation mediated the relationships between POS and affective commitment, organizational spontaneity (helping others and making constructive suggestions), and in-role performance. Moreover, some studies have demonstrated a positive relationship between POS and discretionary safety activities, such as safety voice, defined as any individual communication directed at improving safety (Tucker et al., 2008), safety citizenship behaviors (Mearns and Reader, 2008; Reader et al., 2017), and employee safety involvement (Credo et al., 2010).

Up to now, there has been no study that examines whether felt obligation mediates the relationship between POS and reported discretionary safety behaviors; so, in line with the reasoning of Eisenberger et al., we propose an exploratory hypothesis that:

H2: Felt obligation will mediate the relationship between POS and reported participation in discretionary safety activities.

### Anticipatory Reward as a Mediator

The reciprocity norm has been widely used by organizational support theory; however, SET theory also proposes that actors behave in terms of anticipatory rewards (Homans, 1961; Blau, 1964; Emerson, 1976). Neal et al. (2000) proposed that employee perceptions of their wider organizational environment would provide a context within which specific evaluations of the importance of safety are made. They observed that employee perceptions of their wider organizational context, including supportive leadership, appraisal and recognition, predicted their perceptions of the approach the management took toward safety. Laurent et al. (2018) also reasoned that job resources would provide a similar evaluative context for predicting employee perceptions of management approach to safety. They found that employee perceptions of their jobs along dimensions of perceived support, decision latitude, and job quality predicted their perceptions of management commitment to safety. Notable is that perceptions concerning safety measured by Neal et al. and Laurent et al. are considered to inform employees of expectations regarding organizational approval or disapproval for their safety behaviors, that is, informing them of behavior-outcome expectancies (Zohar, 2008). Thus, perceptions that management is committed to safety are hypothesized to entail an instrumental motivational process (Chmiel and Hansez, 2016), and may directly affect safety behaviors of employees according to what they think is expected of them and the rewards they may expect by behaving accordingly. For example, through interviews, Dilda et al. (2009) identified that the main reason given by oil and gas workers for engaging in safety citizenship behaviors was their perception that this was what was expected of them. In line with this reasoning, both Neal et al. (2000) and Laurent et al. (2018) found that such perceptions predicted employee reports of participation in discretionary safety activities.

In short, perceptions that management is committed to safety entail anticipatory reward from a SET perspective. Similarly, the concept of POS as an evaluative context includes the idea of reward. Eisenberger et al. (2001) proposed that “according to OST, POS meets socioemotional needs, provides assurance that aid will be available when needed, and indicates the organization's readiness to recompense efforts made on its behalf” (p. 42). We reason, therefore, that POS may be considered by employees as a favorable general organizational context within which to evaluate the approach of their organization to safety. A positive view that their organization values their contributions and cares for their well-being will lead to the perception among employees that their organization has a positive approach toward safety concerns that could affect them, and the expectation that behaviors around safety will receive approval. Consistent with this view, POS has been shown to be positively related to such perceptions (Gyekye and Salminen, 2007; DeJoy et al., 2010). Gyekye and Salminen (2007) showed that workers perceiving high organizational support also perceived more commitment and contribution of their management to safety, such as more rewards, than their colleagues perceiving low support. We expect, therefore, that employee perceptions of the commitment of their management to safety will be predicted by POS, which in turn will predict employee reports that they participate in discretionary safety activities.

H3: Perceived management commitment to safety will mediate the relationship between POS and reported participation in discretionary safety activities.

### Job Engagement as an Alternative Mediator

We have outlined in the previous sections the expectations for the main two motivators entailed from a social exchange perspective, thereby examining for the first time the role felt obligation may play in the relationship between POS and reported participation in discretionary safety activities. However, SET and OST suggest that an effect of high-quality exchanges is the willingness of employees to base affective commitment and work effort on the favorableness of treatment received from the organization (Eisenberger et al., 2001). Consistent with this view Orpen (1994) showed a link between POS and effort and performance, and Eisenberger et al. (2001) showed that the relationship between POS and in-role performance was mediated by felt obligation. Of interest, therefore, is whether work-related effort and involvement could be a potential alternative mediator of the relationship between POS and discretionary safety behaviors.

Job engagement is a measure of the effort and involvement with which employees approach their work, and has been shown to predict both in-role and extra-role performance (c.f., Bakker et al., 2004; Bakker and Demerouti, 2007). Saks (2006) has shown further that POS predicted job engagement, and Caesens et al. (2016) showed that job engagement mediated the relationship between POS and proactive behaviors oriented toward the organization. Thus, we explore the possibility that job engagement acts as an alternative mediator between POS and participation in discretionary safety activities.

H4: Job engagement will mediate the relationship between POS and reported participation in discretionary safety activities.

As noted above, Eisenberger et al. (2001) found that felt obligation mediated the relationship between perceived organizational support and in-role performance. Moreover, in a model testing antecedents and consequences of job engagement, and showing that POS was a significant predictor of POS, Saks (2006) argued that workers may engage more in their job because they feel the obligation to do so, as a way to repay their organization for the positive treatment they receive. Thus, we explore whether felt obligation acts as a mediator between POS and job engagement, and expect:

H5: Felt obligation will mediate the relationship between POS and job engagement.

### Employee Perspectives on Participation in Discretionary Safety Activities as Part of Their Job (SCRDs)

Lastly, we turn to the question of whether employees may reciprocate by regarding discretionary safety behaviors to be part of their role or job as a result of feeling valued and cared for by their organization. Such perspectives have been called safety citizenship role definitions (SCRDs). Their perspective is of interest because previous research has shown that employees who say they regard such discretionary behaviors as part of their role are more likely to report they behave accordingly (Chmiel et al., 2017) and are rated by their team leaders as more likely to behave accordingly (Hofmann et al., 2003). Interestingly, Hofmann et al. (2003) observed that employee perspectives were related to the quality of exchanges with their leaders, so it is reasonable to explore whether the relationship between POS and reported participation is mediated by safety role definitions: We hypothesize, therefore, that:

H6: SCRDs will mediate the relationship between POS and reported participation in discretionary safety activities.

Since changing their perspective of what they regard as included in their job (i.e., SCRDs) is a means by which employees can reciprocate their beneficial treatment, we reason that they are likely motivated by the motivators that predict reports of participation in discretionary safety behaviors. Their perspective could be a result of feeling obliged or being expected and rewarded to consider discretionary safety behaviors as part of their job. However, Laurent et al. (2018) found no link between anticipatory reward and safety role definitions, so it is unlikely that expectation and reward play a role in explaining them. We explore the possibility, though, that felt obligation acts as a mediator between POS and SCRDs, with the expectation that:

H7: Felt obligation will mediate the relationship between POS and SCRDs.

Finally, we note that putting an extra effort and commitment into work allows for the possibility that employees could elect to incorporate additional discretionary activities into their job role and come to regard them as part of their job. We, therefore, consider this possibility by examining a link between job engagement and SCRDs. Laurent et al. (2018) did observe a positive relationship between job engagement and SCRDs, so we expect that:

H8: Job engagement will mediate the relationship between POS and SCRDs.

**Figure 1:**
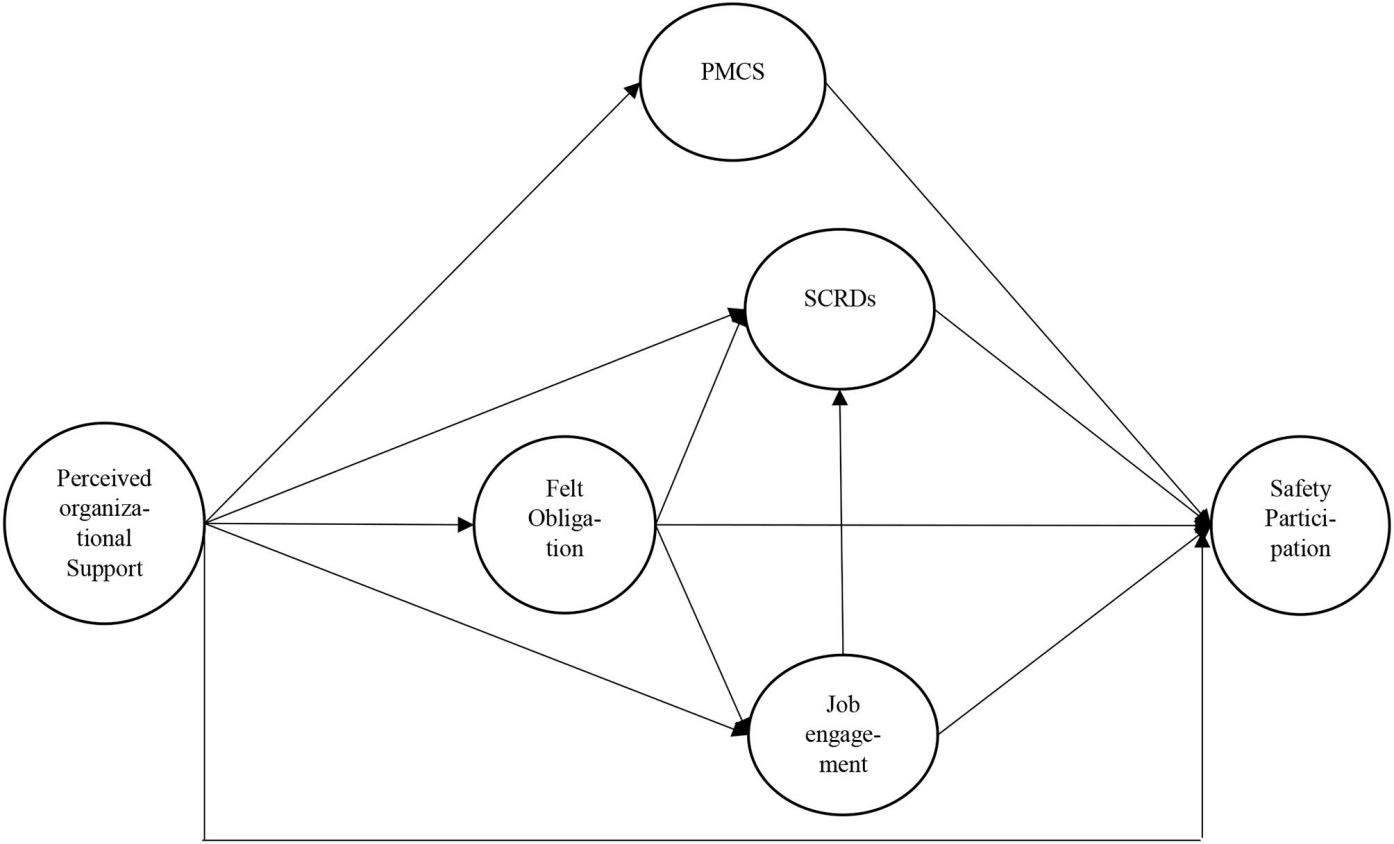
Hypothetical model. PMCS, perceived management commitment to safety; FO, felt obligation; SCRDs, safety citizenship role definitions; Safety participation, reported participation in discretionary safety activities.

The hypotheses are represented in the research model presented in Figure 1. We also included a direct path between POS and safety participation reports to examine whether processes other than those described were involved.

## Method

### Research Approach

For this study, we used a deductive research approach. We developed a theoretical model, on the basis of the hypotheses, emerging from the gaps we identified in the literature and from the questions that remained open from previous studies in the occupational safety research field.

### Sample and Procedure

Self-reported questionnaires were administered to employees in a Belgian public company involved in the production and distribution of safe drinking water, and in waste water treatment. Indeed, regarding the research objectives, we looked for companies responding to the “high-risk” criteria. We consulted the list of Belgian companies classified as SEVESO (*SEVESO Directive 2012/18/EU*), and found that some sites of this public water company responded to the criteria. Data have been collected in the context of safety training sessions organized for blue collar workers by the safety advisor from the company, and in the presence of the researcher conducting the survey, between September and October 2016. The blue-collar workers who took part in this training were free to participate or not to the survey. A total of 553 workers, from the 777 blue-collars workers employed in the company, completed the questionnaires, which corresponded to a response rate of 71%. After removing from the sample population having not responded to at least half of the questions included in the questionnaire, the final sample comprised 536 respondents. All the participants were men, and the mean age was 45 years old. The majority of workers had more than 11 years of job tenure (66.17%). Finally, the majority of workers had no collaborator under their responsibilities (62.84%), i.e., they have no subordinates to supervise.

### Measures and Instrument Development

All the scales we used in this study were previously used and validated. The authors of these scales were contacted and were asked for permission. As the study was conducted in a French and Dutch-speaking context, following the procedure recommended by Brislin (1980), all the measures were translated from English into French/Dutch by a first translator and then independently back-translated into English by other translators. If discrepancies existed between the original and the destination languages, adjustments were made based on a discussion with the translators.

#### Perceived Organizational Support

Perceived organization support was measured using three items from the adaptation of Stinglhamber and Vandenberghe (2003) (α = 0.74) of (1986) Survey of Perceived Organizational Support by Eisenberger. Example items is “*My organization strongly considers my goals and values*.” The respondents were asked to rate their agreement with each item on a 4-point Likert scale.

#### Perceived Management Commitment to Safety

Eight items by Hansez and Chmiel (2010) were used to assess perceived management commitment to safety. These authors found a good Cronbach alpha (α = 0.87). Example item is “*My management has a positive attitude toward safety*.”

#### Felt Obligation

Felt obligation was measured with the seven items developed by Eisenberger et al. (2001). They found a good Cronbach alpha (α = 0.88). Example item is “*I feel a personal obligation to do whatever I can to help my organization to achieve its goals*.”

#### Safety Citizenship Role Definitions

Safety citizenship role definitions were measured with four items from the SCRD scale of Hofmann et al. (2003). The original scale, comprising 27 items, showed good reliability (α = 0.98). We used the items related to initiating safety-related change and the item concerning volunteering safety committees. The items asked the respondents about how much of the described behaviors they believe are part of their job or above and beyond their job responsibilities. The items are “*Trying to change the way the job is done to make it safer*,” “*Volunteering for safety committees*,” “*Trying to improve safety procedures*,” and “*Trying to change policies and procedures to make them safer*.” These items were responded to using a 4-point Likert scale: part of my job (1), somewhat above and beyond my job (2), largely above and beyond my job (3), and definitely above and beyond my job (4). The items were reverse coded, so a higher score indicates that employees considered a discretionary activity to be more part of their job.

Job engagement was measured with 11 items from the Positive and Negative Occupational States Inventory (PNOSI, Barbier et al., 2009). The positive occupational scale we used showed good reliability in the validation paper (α = 0.88). Example item is “*Once I'm at work, I feel more focused*.” The respondents were asked to rate how they felt at work over the last 7 days, on a 4-point Likert scale ranging from 1 (Never or rarely) to 4 (Always or almost always).

Safety participation was measured with the four items developed by Neal et al. (2000) who found good reliability (α = 0.89). Example item is “*I voluntarily carry out tasks or activities that help to improve workplace safety*.”

For all the scales except POS, job engagement and SCRDs, the respondents were asked to rate their agreement with each item on a 5-point Likert scale.

## Results

### Data Analysis

Structural equation modeling analyses were performed using MPlus6. Data were analyzed following a recommended two-step process (Anderson and Gerbing, 1988). First, the measurement models were assessed through a series of confirmatory factor analyses (CFAs) to evaluate the independence of constructs examined in this study. Second, we proceeded with the assessment of the hypothesized structural relationships among latent variables. To limit the number of parameters to be estimated, the number of items was reduced to three for each factor, combining items with the highest and lowest loadings on each latent factor into one indicator (Landis et al., 2000). This parceling strategy preserves the common construct variance while minimizing unrelated specific variance (Little et al., 2013).

Furthermore, to be able to confirm the mediation hypotheses, bootstraps were used in MPlus (on the basis of the model without interaction term) to estimate indirect effects.

### Covariates

Using the full partial covariate effects (Little, 2013), two socio-demographical variables were significantly related with the constructs of the model and were consequently included as covariates. More precisely, the “age” variable was significantly related to safety participation and SCRDs, and the “hierarchical responsibilities” variable was significantly related to felt obligation, SCRDs, and job engagement.

### Measurement Models

As presented in Table 1, the distinctiveness between the variables included in this study is tested through the comparison of several nested models (Bentler and Bonnett, 1980). First, the fit of the hypothesized six-factor model comprising POS, PMCS, felt obligation, SCRDs, job engagement, and safety participation were examined. The results indicated that this hypothesized measurement model fitted the data well [χ^2^(df) = 243.77(120), CFI = 0.97, NNFI = 0.96, RMSEA = 0.04]. A series of five-factor models obtained by combining factors that could share similarities, and a one-factor model, were also tested. A chi-square difference test was performed to compare the nested models (Bentler and Bonnett, 1980). The results indicate that the six-factor model was significantly superior to all the more constrained models. Consequently, this six-factor model was used to test the hypotheses.

**Table 1:** Fit indices for the measurement models.

**Models**	***df***	**χ^2^**	**RMSEA**	**SRMR**	**CFI**	**NNFI**	**Δχ^2^ (Δdf)**
6-factor model	120	243.77	0.04	0.04	0.97	0.96	–
5-factor model (combining PMCS and POS)	125	695.97	0.09	0.06	0.84	0.80	452.20 (5)^***^
5-factor model (combining POS and FO)	125	660.41	0.09	0.08	0.85	0.81	416.64 (5)^***^
5-factor model (combining FO and Job Engagement)	125	571.40	0.08	0.07	0.87	0.85	327.63 (5)^***^
5-factor model (combining FO and SCRDs)	125	713.78	0.09	0.10	0.83	0.80	470.01 (5)^***^
5-factor model (combining FO and Safety Participation)	125	429.85	0.07	0.06	0.91	0.89	186.08 (5)^***^
5-factor model (combining SCRDs and Safety Participation)	125	542.34	0.08	0.09	0.88	0.86	298.57 (5)^***^
1-factor model	136	2225.77	0.17	0.15	0.41	0.33	1982.00 (16)^***^

*N = 536; PMCS, perceived management commitment to safety; POS, perceived organizational support; χ^2^, minimum fit function chi-square; df, degrees of freedom; RMSEA, root-mean-square error of approximation; SRMR, standardized root mean square residual; CFI, comparative fit index; NNFI, non-normed fit index; Δχ^2^, chi-square difference tests*.

*** *p < 0.001*.

### Relationship Among Variables

#### Descriptive Statistics

Table 2 displays the means, standard deviations, alpha levels, and correlations among the variables. It can be noticed that all Cronbach's alphas were acceptable, ranging from 0.66 to 0.93. Concerning the correlations, they were all significant, except the correlation between SCRDs and job engagement (0.06, *p* > 0.05). All correlations were comprised between 0.9, *p* < 0.05 and 0.38, *p* < 0.001, meaning the correlations were not too high, which confirms the results of the CFA, showing the distinctiveness of the variables. As proposed by the SPSS program we used, missing data were replaced by the mean.

**Table 2:** Descriptive statistics and inter-correlations among the variables.

	**Variables**	***M***	***SD***	**1**	**2**	**3**	**4**	**5**	**6**
1	Perceived Organizational Support	2.45	0.70	0.83					
2	Perceived Management Commitment to Safety	3.83	0.58	0.38^***^	0.84				
3	Felt Obligation	3.49	0.58	0.23^***^	0.13^**^	0.74			
4	Safety Citizenship Role Definitions	2.94	0.78	0.17^***^	0.09^*^	0.18^***^	0.80		
5	Job Engagement	2.61	0.54	0.27^***^	0.10^*^	0.26^***^	0.06(*ns*)	0.81	
6	Safety Participation	4.01	0.50	0.20^***^	0.20^***^	0.25^***^	0.28^***^	0.22^***^	0.66

*N = 536. Correlations among variables are provided below the diagonal, and Cronbach's alphas are provided on the diagonal*.

* *p < 0.05*,

** *p < 0.01*,

*** *p < 0.001*.

#### Structural Equation Modeling

The model was tested by structural equation modeling. Table 3 presents fit indices for the hypothesized structural model and the alternative model. The hypothesized model fit the data reasonably well, as indicated by the following indices: χ^2^ (152) = 268.57, CFI = 0.97, NNFI = 0.96, RMSEA = 0.04. This model was compared with a nested model, adding direct paths from PMCS to SCRDs (alternative model 1). The χ^2^ difference between these models was not significant, showing that the hypothesized model was the best.

**Table 3:** Fit indices for structural equation modeling (SEM).

**Models**	***df***	**χ^2^**	**RMSEA**	**SRMR**	**CFI**	**NNFI**	**Δχ^2^ (Δdf)**	**Model comparison**
Hypothesized model	152	268.27	0.04	0.04	0.97	0.96	–	
Alternative model 1 (hypothesized+ pathbetween PMCS and SCRDs)	151	268.21	0.04	0.04	0.97	0.96	0.06 (1) ns	Hypothesized vs. Alternative 1

*N = 536; χ^2^, minimum fit function chi-square; df, degrees of freedom; RMSEA, root-mean-square error of approximation; SRMR, standardized root mean square residual; CFI, comparative fit index; NNFI, non-normed fit index; Δχ^2^, chi-square difference tests. ns, not significant*.

**Figure 2:**
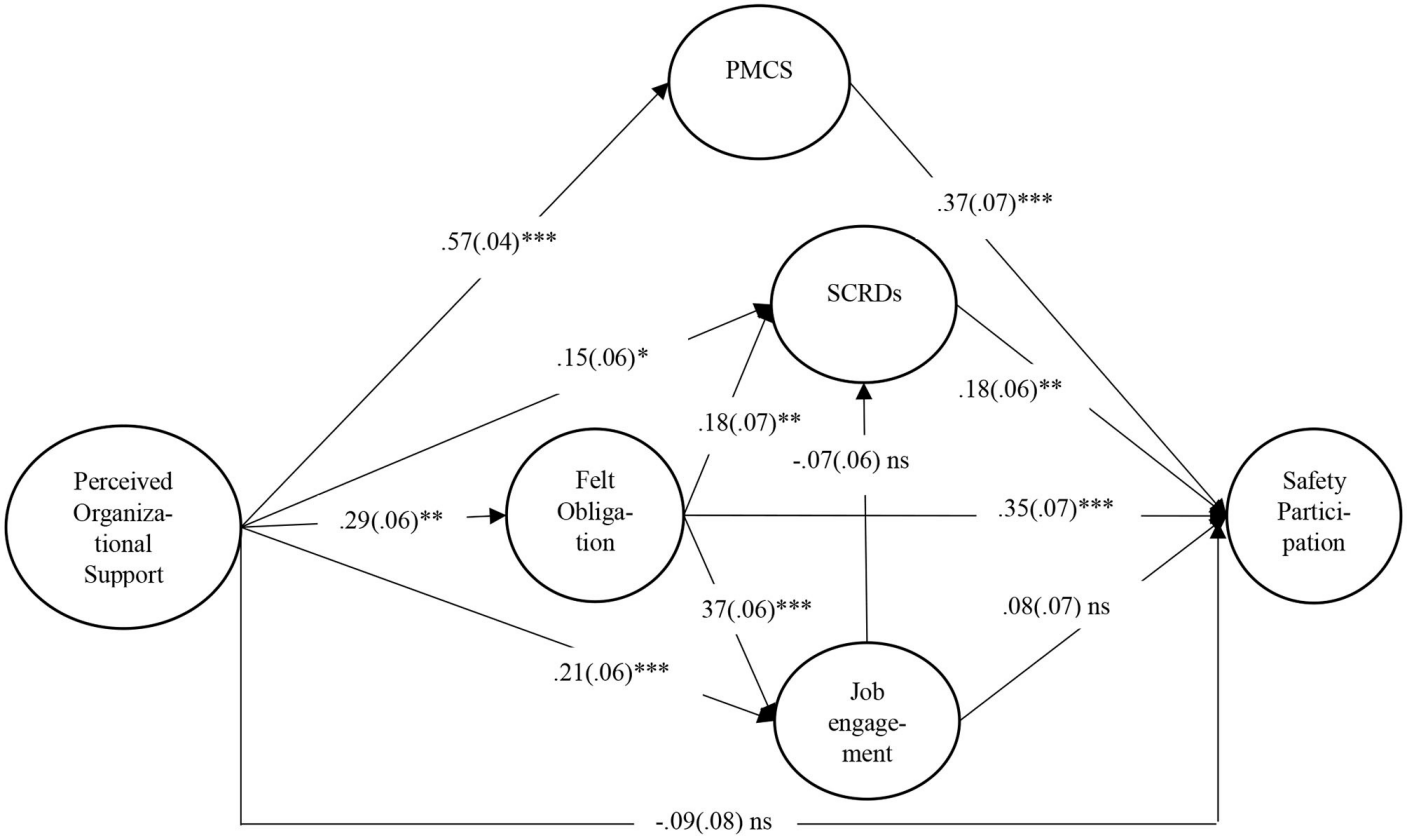
Final model. *N* = 536. Standardized coefficient paths. *p < 0.05, **p < 0.01, ***p < 0.001. Standard error within parentheses. PMCS, perceived management commitment to safety; SCRDs, safety citizenship role definitions.

For ease of presentation, the structural model is presented in Figure 2 rather than the full measurement model, and the covariates are not represented. Only standardized parameters estimates are shown in Figure 2.

To be able to confirm the mediation hypotheses, bootstrap analyses were performed to estimate indirect effects. This method generates a sampling distribution for the indirect effect empirically by repeatedly estimating the indirect effect after sampling from the existing data set with replacement, and estimating the model in each resample (Preacher and Hayes, 2008). Table 4 presents the bootstraps results. The correlation matrix shows that there is a significant relationship between POS and reported participation in discretionary safety activities, supporting hypothesis 1. However, the model (Figure 2) shows that POS was not directly related to reported safety participation (−0.09, SE = 0.06, *p* = ns). Rather, this relationship is mediated by felt obligation and PMCS. The relationship between POS and felt obligation is significant (0.29, SE = 0.06, *p* < 0.01), as is the relationship between felt obligation and reported safety participation (0.35, SE = 0.07, *p* < 0.001), and the bootstrap analysis shows that this indirect effect is significant [0.15, SE = 0.04, 95% CI (0.069; 0.227)], confirming hypothesis 2. It means that the more workers perceived their organization gave them support, the more they felt obliged to reciprocate this support, leading them to report a greater engagement in safety participation behaviors.

**Table 4:** Bootstrap.

	**Bootstrapping**		**Percentile 95% CI**	
	**Effect**	**SE**	**Lower**	**Upper**
**Indirect effect: x → m → y (simple mediation)**				
Perceived organizational support → felt obligation → safety participation	0.15	0.04	0.069	0.227
Perceived organizational support → SCRDs → safety participation	0.03	0.02	−0.009	0.061
Perceived organizational support → PMCS → safety participation	0.21	0.05	0.108	0.315
Perceived organizational support → felt obligation → SCRDs	0.08	0.03	0.016	0.151
Perceived organizational support → felt obligation → job engagement	0.16	0.03	0.092	0.223
Felt obligation → SCRDs → safety participation	0.03	0.02	0.000	0.063
**Indirect effect: x → m1 → m2 → y (double mediation)**				
Perceived organizational support → felt obligation → SCRDs → safety participation	0.02	0.01	0.000	0.029

Perceived organizational support was also significantly related to PMCS (0.57, SE = 0.04, *p* < 0.001), which in turn was related to reported participation (0.37, SE = 0.07, *p* < 0.001). In other words, the more workers perceived organizational support, the more they expected to be rewarded (PMCS) for their increased participation to safety. The mediating role of PMCS in the relationship between POS and SP was also significant [indirect effect: 0.21, 95% CI (0.108; 0.315)], confirming hypothesis 3.

Perceived organizational support was positively related to job engagement (0.21, SE = 0.06, *p* < 0.001) but job engagement was not related to reported safety participation (0.08, SE = 0.07, ns). This leads us to reject exploratory hypothesis 4. However, as the relationships between POS and felt obligation (0.29, SE = 0.06, *p* < 0.01), and between felt obligation and job engagement are significant (0.37, SE = 0.06, *p* < 0.001), as well as the indirect relationship between POS and job engagement through felt obligation [indirect effect: 0.16, SE = 0.03, 95% CI (0.092; 0.223)], hypothesis 5 can be confirmed. Finally, POS was significantly related to SCRDs (0.15, SE = 0.06, *p* < 0.05), which is in turn related to reported participation to safety (0.18, SE = 0.06, *p* < 0.01). Surprisingly, however, this indirect relationship is not confirmed by bootstrap [0.03, SE = 0.02, CI (−0.009; 0.061)], leading us to reject hypothesis 6. It is worth noting that the possibility of a double mediation effect of felt obligation and SCRDs in the relationship between POS and participation was also not supported. The positive relationship between POS and SCRDs is mediated by felt obligation [indirect effect: 0.08, SE = 0.03, SE = 0.02, CI (0.016; 0.151)], but not by job engagement as the relationship between SCRDs and job engagement is not significant. This allows us to confirm hypothesis 7, but not hypothesis 8.

## Discussion

The findings show a relationship between POS and participation in discretionary safety behaviors. In accordance with social exchange theory, the relationship was mediated by felt obligation and anticipatory reward, as measured by PMCS (H1, H2, and H3 were supported). Equally of note is that job engagement was shown not to act as an alternative mediator (H4 was not supported), although felt obligation did mediate the relationship between POS and job engagement (H5 was supported).

Previous research has shown that the perspectives employees take on whether discretionary safety behaviors are part of their job role or not (i.e., SCRDs) are important in predicting participation, either reported or actual. Although the findings indicate a significant path between POS and SCRDs, and between SCRDs and participation reports, the bootstrap analysis did not support H6, that SCRDs would mediate the relationship between POS and participation reports, as the 95% CI included 0, as did the double mediation effect between POS and participation reports of felt obligation and SCRDs. Felt obligation did mediate the relationship between POS and SCRDs, however, supporting H7, whereas job engagement did not, so H8 was not supported.

This study addressed the question of why employees may choose to participate in discretionary safety behaviors and examined motivators, felt obligation and anticipatory reward, entailed by a social exchange perspective. To the knowledge of the authors, this study is the first to show that felt obligation is involved in predicting reports of participation in discretionary safety behaviors as a result of employees feeling valued and cared for by their organization. Moreover, anticipatory reward, in the form of PMCS, was also involved in mediating the relationship between POS and reported participation in discretionary safety behaviors. Thus, both fundamental motivators advanced by SET are involved in the relationship between employees believing their organization values their contribution and cares about them and their choice to report to take part in discretionary safety behaviors, thereby adding to the understanding of discretionary safety behavior.

Previous research has suggested that job engagement mediates the relationship between POS and in-role performance (e.g., Caesens et al., 2016), so we explored whether this was the case here for discretionary (i.e., extra-role) participation in safety behaviors. We found no significant effect of job engagement on reports of participation, or on the choice of employees to regard such behaviors as part of their role. In contrast, Laurent et al. (2018) found that job engagement did mediate the relationship between job resources and participation reports, and between job resources and SCRDs. Further research is needed to elucidate the reasons for this difference, but one possibility is that it is connected to the provision of resources. In other words, employees may feel more engaged as a result of feeling supported and valued, but need resources, for example greater decision latitude, to turn that engagement into behaviors in addition to those mandated by their job.

Organizational support theory, although rooted in social exchange theory, emphasizes the role played by felt obligation in the relationship between POS and a variety of outcomes. Here, we found that felt obligation mediated the relationship between job engagement and employee perspectives on whether discretionary safety behaviors were part of their role (i.e., SCRDs). It follows from OST, therefore, that both these outcomes are able to be reciprocated by employees as a result of feeling valued and cared for by their organization. These findings extend the previous studies by Saks (2006) and Caesens et al. (2016), especially in providing support for the idea that employees may change their perspective on their role as a result of feeling obliged to their organization as well as becoming more engaged in their work.

Lastly, we turn to the role played by employee perspectives on whether discretionary safety behaviors are part of their job or not. Previous research has shown that SCRDs predict reported or actual participation in such activities. We find a significant path between SCRDs and reported participation consistent with earlier findings. We also find that the path between POS and SCRDs was significant. What we do not find, however, is evidence of a reliable mediation effect of SCRDs between POS and reports. The pattern of results is supported by the findings of Hofmann et al. (2003). They tested whether SCRDs mediated the relationship between LMX and participation, finding that mediation was not supported. Further research will be needed to establish the reliability of mediation, if there is any, but we note that evidence on this issue is sparse. We observe, though, that Laurent et al. (2018) did find support for SCRDs mediating the relationship between job resources and reports of safety participation, highlighting again the possibility that the combined effect of support and resources may be important.

Taken together, these results further the claims for the use of social exchange theory as a framework for understanding work behaviors. Furthermore, the framework suggests how an organization might encourage employees to report that they participate in discretionary safety behaviors to its benefit: value and care for employees and they are likely to reciprocate if the organization values safety, by feeling obliged and anticipating reward. As a bonus, they may also become more engaged in their jobs and change their perspective on their job role *vis a vis* safety.

### Limitations and Future Research Directions

This study comprises cross-sectional self-report data and caution is required when interpreting the findings, since causality cannot be inferred from such data. Future studies are desirable to validate these effects, using longitudinal designs and objective measures.

In addition, the use of self-reported data may lead to common-method variance bias. More precisely, for example, actual participation in voluntary safety activities was not measured, what was measured was self-reported participation in voluntary safety activities. However, this problem was partially addressed, since the results of confirmatory factor analyses indicated that a single-factor model showed poor fit to the data (i.e., Harman's single-factor test; Podsakoff et al., 2003). Future studies should also include objective data and observation of safety behaviors in order to confirm the results.

Another weakness of this study is the relatively low reliability of the safety participation scale. However, although 70 is often considered as the minimum threshold for good reliability, in a study referring to 69 articles published in a single year (2015), Taber (2018) explored how Cronbach's alpha is interpreted in education research. In these reported studies, a 0.66 Cronbach's alpha is qualified as ≪ acceptable ≫, ≪ satisfactory ≫ or ≪ sufficient.≫

An avenue for future studies could be to apply the present model to different dimensions of safety citizenship behaviors. Indeed, safety participation behaviors, by being discretionary, are similar to citizenship behaviors, Hofmann et al. (2003) created the safety citizenship behaviors scale comprising 27 items, classified into six dimensions (i.e., helping, voice, stewardship, whistle-blowing, civic virtue, initiating safety-related change).

Another main limitation is related to the generalizability of the findings. As in all research, caution is required before generalizing the findings to the whole population. Specifically, the research sample only comprised male workers, which did not allow the authors to generalize to female public. However, as socio-demographical variables were controlled, using the full partial covariate effect (Little, 2013), it should not be problematic for conclusions to be drawn.

### Conclusion and Practical Implications

This study used social exchange theory to explain the mechanisms by which POS led workers to engage in safety citizenship behaviors. Specifically, on the one hand, POS elicited instrumental processes, by fostering PMCS of the workers, i.e., a safety-specific signal that rewards can be expected if they behave safely. These perceptions, in turn, led to workers engaging in SP, but only under the condition that the actions and attitudes of their supervisor toward safety were perceived as consistent, i.e., only if they trusted their supervisor. On the other hand, POS also elicited obligation processes, by provoking felt obligation from workers to reciprocate the positive treatment they received, leading them to engage in SP behaviors, directly and indirectly, through defining safety as part of their job role (i.e., SCRDs).

Taken together, these results emphasized the powerful role played by general perceptions of support from the organization in determining positive safety behaviors. However, perceiving general support from the organization and perceiving safety as being valued at the same organizational/managerial level are not sufficient conditions for workers to engage in extra-role safety behaviors. Indeed, it is also necessary that the actions and attitudes of their direct supervisor toward safety be perceived as consistent. In other words, interpersonal trusting relationships must be built between workers and their direct supervisors. Thus, it is important for companies willing to improve the SP of their workers to know the importance of these social exchange processes. First, they should enhance the perceived organizational support of employees. To this end, Eisenberger and Stinglhamber (2011) identified key levers allowing to improve POS: for example, managers could be trained to communicate the voluntary nature of favorable actions and the involuntary nature of unfavorable ones; display sincerity through consistency of discourse and actions; treat employees fairly, respectfully, and courteously; provide meaningful training and developmental programs promoting personal growth, knowledge, and career goals, and promote fairness in administering policies and allocating rewards. Second, besides these non-safety specific aspects necessary to enhance POS, managers could be trained specifically about the importance of their true commitment to safety. To this end, Pedersen and Nielsen (2013) proposed an intervention based on an integrative approach of safety management (DeJoy, 2005) comprising problem-solving process, taking the form of practical workshops with workers and managers and culture-change process, and taking the form of individual coaching sessions with managers that focus on their crucial role for safety. By fostering management support for safety and the involvement of workers in safety, this type of intervention is targeted at improving affective commitment to the organization, and mutual trust and reciprocity between managers and workers, which have been identified in this study as key aspects leading to increased participation to safety.

## Data Availability Statement

The raw data supporting the conclusions of this article will be made available by the authors, without undue reservation.

## Ethics Statement

The studies involving human participants were reviewed and approved by ethical committee of the Faculty of Psychology and Education, University of Liege. Written informed consent for participation was not required for this study in accordance with the national legislation and the institutional requirements. Oral consent were given by each participant during collective sessions, and that their rights were provided into a letter containing the researchers' contact details.

## Author Contributions

JL was the main project leader. She collected and analyzed the data under the supervision of IH. NC has an important conceptual role and took an great part in the redaction of the paper. All authors listed have made a substantial, direct and intellectual contribution to the work, and approved it for publication.

## Conflict of Interest

The authors declare that the research was conducted in the absence of any commercial or financial relationships that could be construed as a potential conflict of interest.

## Publisher's Note

All claims expressed in this article are solely those of the authors and do not necessarily represent those of their affiliated organizations, or those of the publisher, the editors and the reviewers. Any product that may be evaluated in this article, or claim that may be made by its manufacturer, is not guaranteed or endorsed by the publisher.

## Footnotes

^1^“It's important to note that, in this study, we consider safety participation and safety citizenship behaviors as similar constructs, because they are discretionary and represent safety-specific contextual performance, as opposed to task-related performance (e.g., Borman and Motowidlo, 1993; Christian et al., 2009).
